# *In vivo* safety and efficacy testing of a thermally triggered injectable hydrogel scaffold for bone regeneration and augmentation in a rat model

**DOI:** 10.18632/oncotarget.24813

**Published:** 2018-04-06

**Authors:** Abbey A. Thorpe, Christine Freeman, Paula Farthing, Jill Callaghan, Paul V. Hatton, Ian M. Brook, Chris Sammon, Christine Lyn Le Maitre

**Affiliations:** ^1^ Biomolecular Sciences Research Centre, Sheffield Hallam University, S1 1WB, UK; ^2^ School of Clinical Dentistry, University of Sheffield, S10 2TA, UK; ^3^ Materials and Engineering Research Institute, Sheffield Hallam University, S1 1WB, UK

**Keywords:** mesenchymal stem cell, hydrogel, injectable, bone regeneration, preclinical studies

## Abstract

Bone loss resulting from degenerative diseases and trauma is a significant clinical burden which is likely to grow exponentially with the aging population. In a number of conditions where pre-formed materials are clinically inappropriate an injectable bone forming hydrogel could be beneficial. The development of an injectable hydrogel to stimulate bone repair and regeneration would have broad clinical impact and economic benefit in a variety of orthopedic clinical applications.

We have previously reported the development of a Laponite^®^ crosslinked pNIPAM-co-DMAc (L-pNIPAM-co-DMAc) hydrogel delivery system, loaded with hydroxyapatite nanoparticles (HAPna), which was capable of inducing osteogenic differentiation of mesenchymal stem cells (MSCs) without the need for additional growth factors *in vitro*. However to enable progression towards clinical acceptability, biocompatibility and efficacy of the L-pNIPAM-co-DMAc hydrogel to induce bone repair *in vivo* must be determined.

Biocompatibility was evaluated by subcutaneous implantation for 6 weeks in rats, and efficacy to augment bone repair was evaluated within a rat femur defect model for 4 weeks. No inflammatory reactions, organ toxicity or systemic toxicity were observed. In young male rats where hydrogel was injected, defect healing was less effective than sham operated controls when rat MSCs were incorporated. Enhanced bone healing was observed however, in aged exbreeder female rats where acellular hydrogel was injected, with increased deposition of collagen type I and Runx2. Integration of the hydrogel with surrounding bone was observed without the need for delivered MSCs; native cell infiltration was also seen and bone formation was observed within all hydrogel systems investigated.

This hydrogel can be delivered directly into the target site, is biocompatible, promotes increased bone formation and facilitates migration of cells to promote integration with surrounding bone, for safe and efficacious bone repair.

## INTRODUCTION

Bone loss resulting from degenerative diseases and trauma is a significant clinical burden which is likely to grow exponentially with the aging population [1]. Bone autografts are considered the gold standard treatment for bone repair; however, this is hampered by limited availability and donor-site morbidity [2]. Allografts are more readily available but can result in immunogenicity and poor outcomes [3, 4]. Consequently, there is a growing clinical need for the development of novel synthetic bone grafting materials which can substitute and augment bone effectively [5, 6]. A variety of bone substitute materials including: ceramics [7, 8] and metals [9] have been used therapeutically. However, these are synthesised as pre-formed constructs, and normally implanted by invasive surgery increasing operative risk.

Hydrogels, are a group of biomaterials which may overcome these limitations as they can be administered in a minimally invasive manner and fill complex cavities *in vivo* [10]. Recently Lohmann *et al.,* 2017 reported the use of a 3D architectured hydrogel *in vivo*, within a rat calvarial defect, which displayed significant promise for bone regeneration comparable with autologous bone grafts [11]. However, similarly to the clinical bone grafts that are currently available, this hydrogel was implanted as pre-formed discs [11]. Temperature sensitive hydrogels are attractive since they can be applied as a liquid directly into the defect site, before *in situ* gelation at body temperature [12]. Furthermore, hydrogels enable the incorporation of bioactive factors such as calcium-based minerals to enhance scaffold mineralisation and osteogenicity [13–16]; they also facilitate the delivery of regenerative cells such as mesenchymal stem cells (MSCs) [17–19], the combination of these factors may provide intrinsic osteogenic capabilities [20]. Studies have also shown that substrate stiffness and nanotopography can influence cell attachment, function and differentiation [21–24]. The incorporation and delivery of MSCs within a suitable hydrogel would not only serve to maintain the delivered MSCs at the injection site, but could also promote integration with surrounding bone tissue and provide a micronevrimonent surrounding the cells to modulate their osteogenic differentiation. A major drawback of hydrogels is that they do not possess the mechanical robustness to be used in load bearing applications [25], however their use in a variety of non-weight bearing orthopedic situations could be beneficial particularly where pre-formed materials are clinically inappropriate or challenging. Applications could include: i) augmenting bone around dental implants and for treatment of bone defects in patients with periodontal disease which affects 45% of adults moderately and 5% of adults severely within the UK [26]; ii) increasing bone density in osteoporosis which affects 3 million in the UK [27, 28]; iii) aiding in the repair of non-union fractures which cost the NHS ~£7000-£79,000 per patient [29], finally, iv) such an approach could be utilised to improve fixation of prosthetic joints that have loosened due to osteolysis [30]. In addition, the use of an injectable hydrogel, with intrinsic osteogenic capacity, could be beneficial in certain load bearing bone applications. For example, to improve and accelerate internal bone fixation and integration of bone cages such as those used in clinical practice for intervertebral disc fusion [31]. The development of an injectable hydrogel to stimulate bone repair and regeneration would therefore have broad clinical impact and economic benefit in a variety of orthopedic clinical applications. An ideal injectable bone graft hydrogel, would be one with: low viscosity for minimally invasive delivery; can fill complex voids *in vivo* before rapid *in situ* gelation; be biocompatible and be osteoconductive to promote integration. To date, hydrogel systems incorporated with MSCs have been reported for bone regeneration (Supplementary Table 1); however few of these are injectable and the majority require the addition of growth factors to stimulate osteogenesis, adding both complexity and cost to the treatment strategy [32–36].

We have previously reported the development of a synthetic Laponite^®^ crosslinked pNIPAM-co-DMAc hydrogel (L-pNIPAM-co-DMAc) loaded with hydroxyapatite nanoparticles (HAPna), which induces osteogenic differentiation of MSCs *in vitro*, without the need for osteogenic growth factors [18]. This synthetic hydrogel is clinically appealing for bone repair since it is cytocompatible in the liquid state and therefore enables the safe incorporation and delivery of MSCs. The hydrogel undergoes rapid gelation at body temperature and the synthesis does not require the addition of chemicals for gelation or clean-up [18, 37]. The combination of all of these properties in one hydrogel system is unlike any other hydrogel system for bone repair previously developed. The clinical success of this hydrogel is dependent on the safe delivery of the scaffold and incorporated MSCs; maintenance within the defect site; integration with surrounding bone tissue; and the capacity to stimulate bone repair within an *in vivo* bone defect.

This study investigated the biocompatibility *in vivo* following rat subcutaneous implantation and biocompatibility and efficacy following injection into a rat femur defect model. L-pNIPAM-co-DMAc hydrogels with and without rat MSCs and HAPna were investigated to test the hypothesis that the delivery of MSCs within L-pNIPAM-co-DMAc, with incorporated HAPna, would aid scaffold integration as well as promote and accelerate bone healing.

## RESULTS

### *In vivo* safety: subcutaneous implantation

#### Gross examination of organs and implantation site

**Figure 1:**
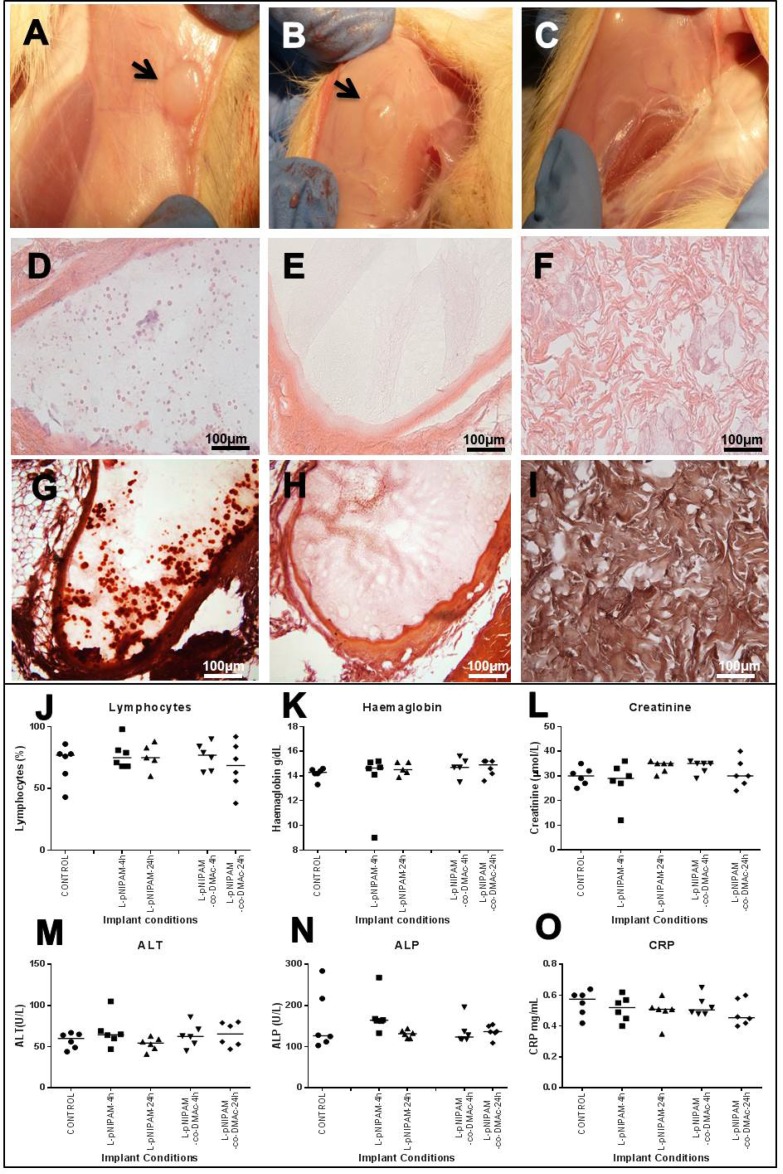
*In vivo* safety analysis following 6 weeks (**A**, **B**) Macroscopic images following 6 weeks after subcutaneous implantation of L-pNIPAM-4h hydrogel (A) and L-pNIPAM-co-DMAc-4h hydrogel (B). Black arrows indicate hydrogel encapsulated by the surrounding tissue. (**C**) Macroscopic image of sham operated control following 6 weeks. (**D**, **E**) haematoxylin and eosin (H&E), of (D) L-pNIPAM-4h hydrogel and (E) L-pNIPAM-co-DMAc-4h hydrogel following 6 weeks. (**F**) H&E of sham operated control following 6 weeks. (**G**) Representative microscopic image stained with alizarin red (AR) demonstrating where calcium deposits were observed within L-pNIPAM-4h hydrogel region. (**H**) Representative microscopic image stained with AR demonstrating where calcium deposits were not observed within L-pNIPAM-co-DMAc-4h hydrogel region. (**I**) AR staining of sham operated control. *Scale bar 100 μm*. (**J**–**O**) Blood and biochemistry analysis. Graphs shown include: lymphocytes, haemoglobin, creatine, alanine transferase (ALT), alkaline phosphatase (ALP) and C-reactive protein (CCRP).

All animals, in all experimental groups and sham operated controls, survived the subcutaneous implantation surgery and 6 weeks postoperative course without any surgery-related or implantation-related complications. The animal weight was recorded before surgery and immediately following sacrifice. Overall the weight of all animals increased slightly indicating good post-operative development and healthy status of all animals (data not shown). Following animal sacrifice, the liver, kidney, testes and lymph nodes were extracted; no gross evidence of infection or organ toxicity was observed in any animal (data not shown). Gross examination of implantation sites demonstrated encapsulation of hydrogel constructs by surrounding tissues (Figure 1A, 1B). No gross differences were observed between hydrogel formulations or sham operated animals (Figure 1A–1C). The tissue surrounding and encapsulating the hydrogel was macroscopically healthy with no signs of infection, inflammation or vascularisation (Figure 1A, 1B).

#### Histological evaluation of organs and implantation site

Histopathological assessment of the implantation site was performed to investigate hydrogel integrity and local tissue response following subcutaneous implantation of hydrogel scaffolds (Figure 1D–1F). All hydrogels remained in place where implanted, with no evidence of angiogenesis, granulation tissue or inflammatory response (Figure 1D, 1E). Hydrogels were surrounded by fibrous connective tissue with no evidence of hydrogel degradation (Figure 1D, 1E). Calcium deposition, confirmed via alizarin red staining, was observed within the hydrogel region of 10 animals (Figure 1G–1I, and Supplementary Table 2), independent of which hydrogel formulation was implanted (Figure 1G–1I and Supplementary Table 2). Haematoxylin and eosin stained liver, kidney, testes and lymph node sections displayed normal histological appearance with no evidence of infection or toxicity in any animal (Supplementary Figure 1).

#### Blood and biochemistry analysis

Blood samples were extracted from animals following sacrifice and full blood count, differential blood count and serum biochemistry was performed (Figure 1J–1O). No significant differences in haematological and serum biochemistry parameters were seen between any animals in any test group compared to sham operated controls, indicating no signs of systemic inflammatory response or organ toxicity (Figure 1J–1O).

### *In vivo* efficacy: femur defect model in 10–12 weeks old male Wistar rats

All animals survived surgery and the 4 week postoperative course without complications. The average weight of all animals increased slightly indicating good post-operative development and healthy status of animals. Furthermore no gross or histological evidence of infection or organ toxicity was observed in any animal.

#### Micro CT analysis of femur defect region in young male rats

**Figure 2:**
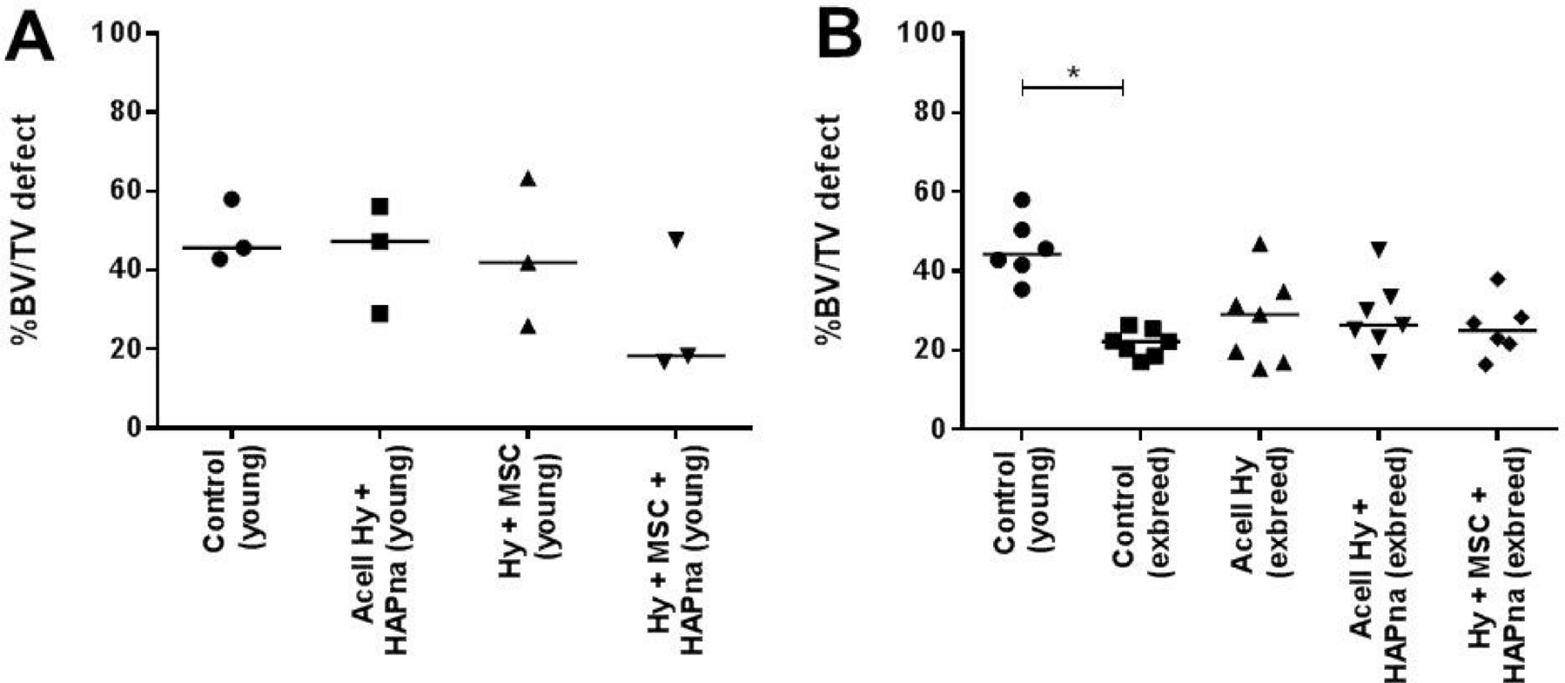
Bone volume (%) assessed using Micro-CT (**A**) Pilot study with 10–12 week (young) male white Wistar rats (*n* = 3). (**B**) Control 10–12 week (young) male white Wistar rats (*n* = 6), compared with older (>7 months) exbreeder female white Wistar rats (*n* = 7). ^*^ indicates statistical significance (*P* ≤ 0.05).

Percentage bone volume (% bv), as an end point measure of bone formation within the femur defect region, was assessed using Micro-CT (Figure 2A). No significant difference in % bv was observed in young male Wistar rats between sham operated animals and hydrogel injected groups (Figure 2A). However a decreasing trend in the % bv was observed in young male Wistar rats where L-pNIPAM-co-DMAc (±HAPna) incorporated with MSCs had been injected (Figure 2A).

#### Histological evaluation of femur defect region in young male rats

**Figure 3:**
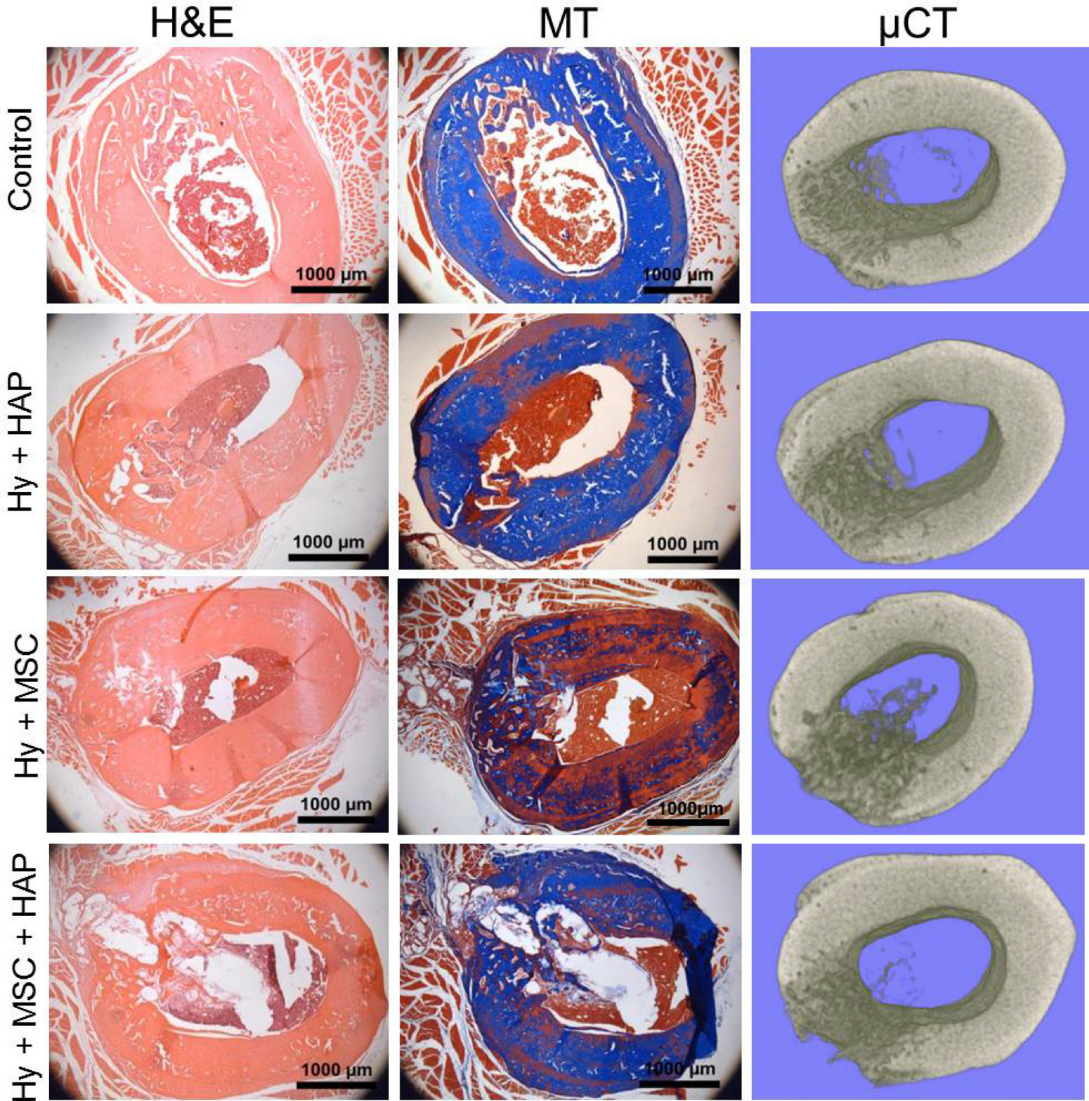
*In vivo* rat femur defect study in 10–12 week old male white Wistar rats H&E and Masson's trichrome as well as 3D Micro-CT reconstructed images of the femur defect site following 4 weeks repair time. *Scale bar 1000 μm.*

Histopathological analysis of the femur defect site in young rats was performed to assess the bone healing response and local tissue response. All three sham operated controls displayed almost complete healing, with newly regenerated immature bone matrix found within the defect region (Figure 3). L-pNIPAM-co-DMAc was observed within the defect region in all animals where it had been injected regardless of whether HAPna and/or MSCs were incorporated (Figure 3). L-pNIPAM-co-DMAc integrated with the surrounding bone tissue with no evidence of fibrous encapsulation (Figure 3). No histological evidence of toxicity or inflammatory response were observed (Figure 3). In general where L-pNIPAM-co-DMAc was injected, regardless of whether HAPna or MSCs were incorporated, the regeneration of new bone matrix was evident in association with the hydrogel (Figure 3). However the hydrogel was seen to disrupt the cortical plate, delaying healing in comparison to sham operated controls (Figure 3). Where acellular L-pNIPAM-co-DMAc with HAPna was injected, the defect region healed well, with bone formation observed in association with the hydrogel and infiltration of numerous osteoblast like cells (Figure 3). Where L-pNIPAM-co-DMAc with incorporated MSCs (±HAPna) had been injected, the healing response was histologically less effective, with large voids showing no evidence of newly regenerated bone matrix, present in the defect region 4 weeks post implantation (Figure 3).

### Micro CT analysis of femur defect region in young vs aged sham operated controls

To test the hypothesis that: aged (>6 months) female exbreeder rats would have a reduced or delayed healing capacity, Micro-CT analysis of the femur defect after 4 weeks was performed on sham operated controls in young males (*n* = 7) and aged female ex-breeder Wistar rats (*n* = 7) (Figure 2B). One young control was excluded due to pre-existing femur pathology. The % bv within the femur defect site of young male Wistar rats in the sham operated control group was significantly higher (*P* = 0.0002) than that of the aged female exbreeder rats 4 weeks post operation (Figure 2B).

### *In vivo* efficacy: femur defect model in aged (>6 months) female ex-breeder Wistar rats

All aged female ex-breeder Wistar rats in the different hydrogel scaffold groups and sham operated control group survived surgery and the 4 weeks postoperative course. One animal injected with L-pNIPAM-co-DMAc +MSC +HAPna, was excluded as no visible defect following 4 weeks could be seen using Micro-CT or histological analysis, as such the region of interest could not be objectively selected.

#### Micro CT analysis of femur defect region in aged (>7 months) female ex-breeder Wistar rats

No significant difference in the % bv was observed between ex-breeder female Wistar rats in the hydrogel scaffold injected groups and sham operated control group (Figure 2B).

#### Histological evaluation of femur defect region in female ex-breeder rats

**Figure 4:**
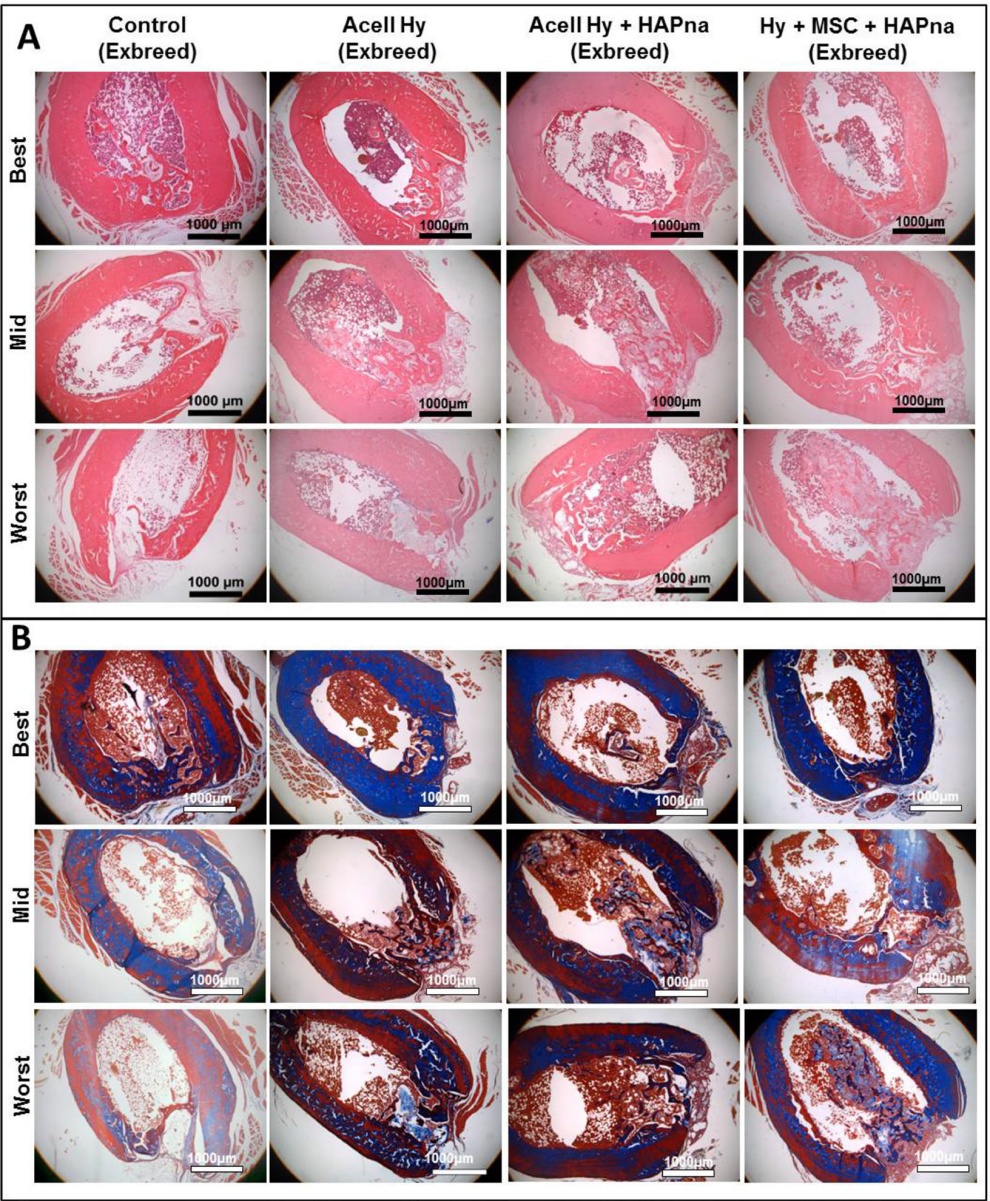
Histological assessment of the defect site after 4 weeks repair time following a non-critical sized defect in the midshaft of the femur in exbreeder female (>6 months old) rats, stained with H&E (**A**) or Masson's trichrome (**B**). Representative images from 6 replicates for each experimental group to demonstrate the best, mid and worst bone repair observed from independent pathological assessment. *Scale bar: 1000 μm.*

Within the sham operated controls, two animals displayed effective healing of the femur defect; whilst fibrous tissue was observed overlying the defect region with little histological evidence of bone healing in the remaining four animals. In general where L-pNIPAM-co-DMAc was injected, regardless of whether HAPna or MSCs were incorporated, the hydrogel integrated with the surrounding bone tissue with no evidence of fibrous encapsulation (Figure 4A). No histological evidence of toxicity or inflammatory response towards the injected hydrogels was observed in any animal (Figure 4A). L-pNIPAM-co-DMAc was observed within the defect region in all animals where it had been injected (Figure 4A). Bone regeneration was confined to the bone cortex region in sham operated controls. However collagen deposition, as identified via Masson's trichrome staining, and newly regenerated bone matrix, extended past the femur cortex and into the bone marrow space where the hydrogel was injected (Figure 4A, 4B). The healing response between sham operated controls and hydrogel injected animals was found to be histologically distinct. Within sham operated controls, bone regeneration was initiated from the cortical plate around the periphery of the defect region and healed towards the centre (Figure 4A). In contrast where L-pNIPAM-co-DMAc had been injected, the bone repair was initiated in the centre of the defect region in direct association with the injected hydrogel (Figure 4A). Where acellular L-pNIPAM-co-DMAc, without HAPna, was injected, 5/7 animals displayed effective healing across the entire width of the defect region. However hydrogel was also located outside the defect region which was not associated with bone remodelling (Figure 4A). Where L-pNIPAM-co-DMAc + HAPna was injected, both with and without incorporated MSCs, the hydrogel was observed within the centre of the defect region in all animals and was found to be associated with newly regenerated bone matrix (Figure 4A). No histological evidence of enhanced repair with the incorporation of MSCs within L-pNIPAM-co-DMAc + HAPna was observed in comparison to the injection of acellular L-pNIPAM-co-DMAc + HAPna (Figure 4A).

#### Assessment of collagen deposition within the femur defect within aged female ex-breeder Wistar rats

**Figure 5:**
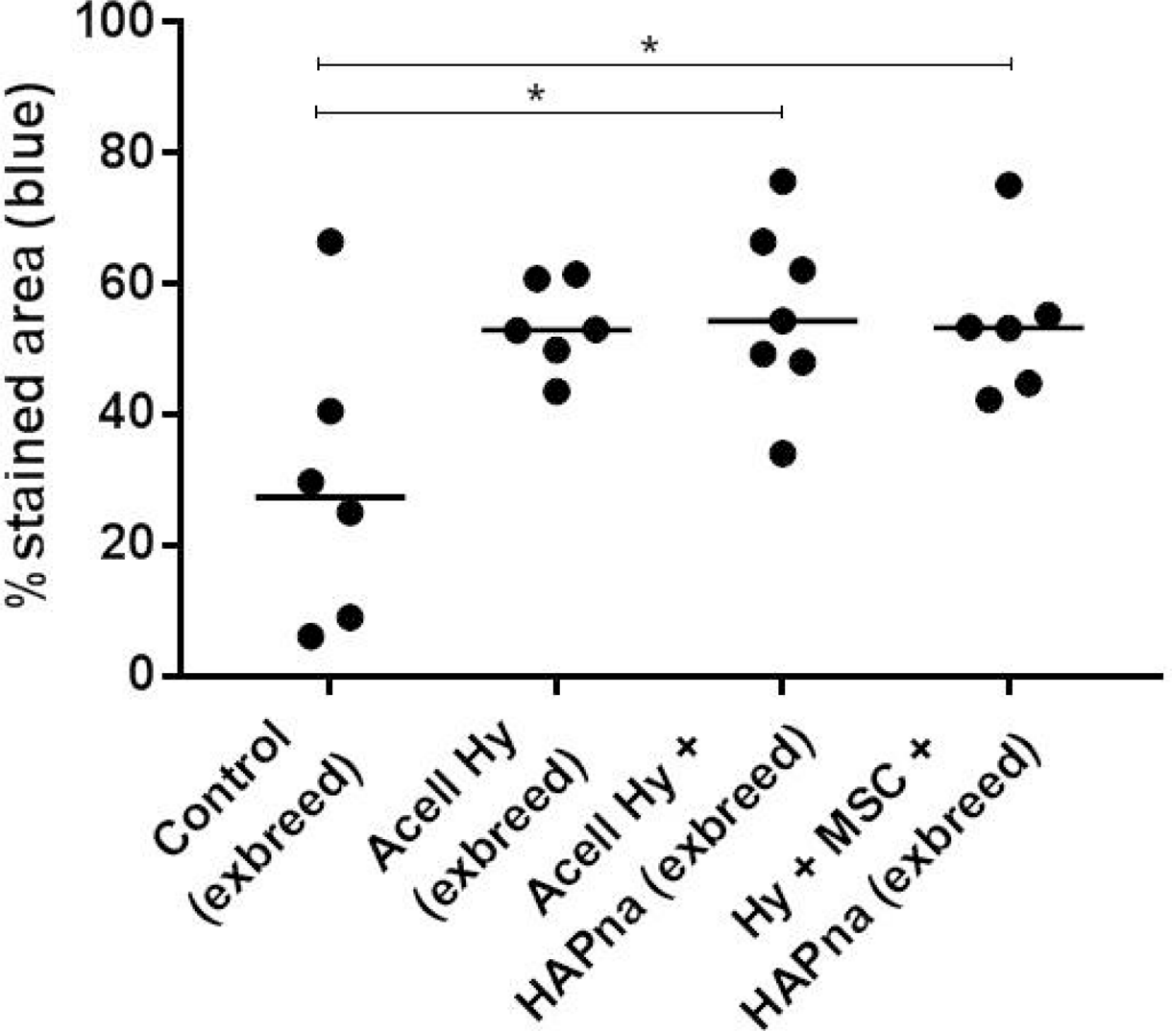
Percentage area of collagen staining (blue) within the femur defect site from histological sections stained with Masson's trichrome, in exbreeder female (>6 months old) rats ^*^ indicates statistical significance *(P = ≤ 0.05).*

Masson's trichrome staining, to assess collagen deposition as an early matrix marker of bone formation, was performed on tissue sections within the femur defect region of aged female Wistar rats (Figures 4B, 5). Cellular and matrix collagen staining in the centre of the defect region was observed histologically in association with L-pNIPAM-co-DMAc in all cases (Figure 4A). Where L-pNIPAM-co-DMAc + HAPna (±MSCs) (*P* = 0.0085 without MSCs, *P* = 0.0197 with MSCs) was injected, the percentage area of collagen staining was significantly increased in the centre of the defect region in comparison to sham operated controls (Figure 5). No significant difference in the percentage area of collagen staining was observed where acellular L-pNIPAM-co-DMAc, without HAPna, was injected in comparison to sham operated controls (Figure 5). No significant difference in the percent area of collagen staining was observed within the defect area between acellular L-pNIPAM-co-DMAc+HAPna with and without incorporated MSCs (Figure 5).

#### Immunohistochemistry evaluation of the femur defect within aged female ex-breeder rats

**Figure 6:**
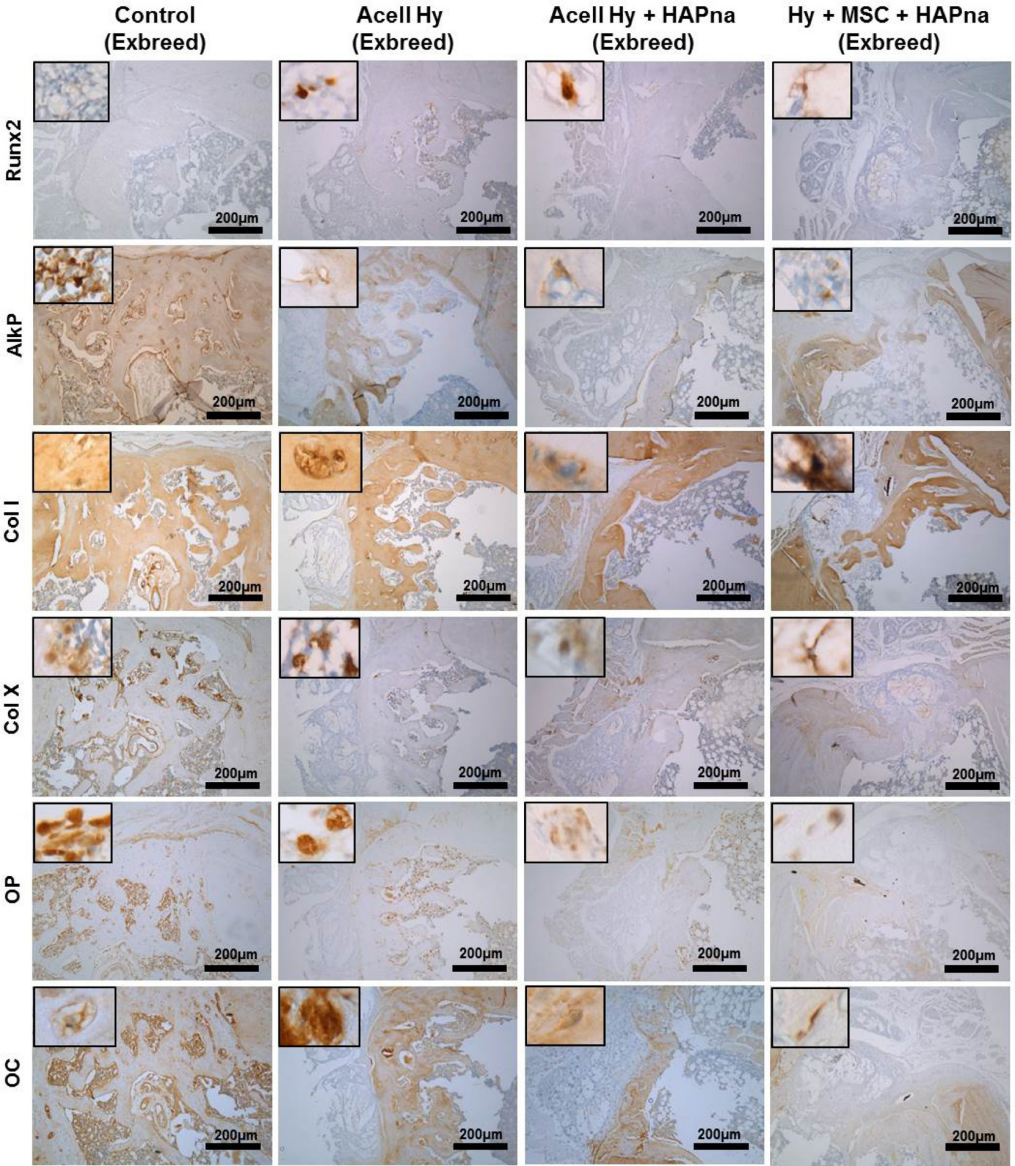
Immunohistochemistry assessment of the defect site in exbreeder female (>6 months old) rats Scale bar: 200 μm.

Immunohistochemistry was utilised to determine protein expression for the macrophage marker: CD68; the early bone markers: runx2 and alkaline phosphatase; the bone matrix markers: collagen type I and collagen type X; as well as the late bone markers osteopontin and osteocalcin to assess the bone healing response within the rat femur defect model following 4 weeks repair time (Figure 6). The macrophage marker (CD68), was only expressed by a few cells within the defect region of both sham operated control and hydrogel injected animals with no difference in expression observed between the different experimental groups (Supplementary Figure 2). The osteoblast specific transcription factor runx2 was expressed by cells within the defect region in all animals, however increased immunopositivity for runx2 were observed in L-pNIPAM-co-DMAc injected defects in comparison to sham operated controls (Figure 6). The highest immunopositivity for runx2 was observed in animals where L-pNIPAM-co-DMAC with incorporated MSCs was injected (Figure 6). Immunopositive cellular and matrix staining for alkaline phosphatase and collagen type I, both early markers of bone regeneration, were highly expressed across the entire width of the defect region in 2/6 sham operated controls, where effective healing occurred (Figure 6). However, in the remaining 4/6 animals, where incomplete healing occurred, limited matrix immunopositivity for alkaline phosphatase and collagen type I were observed (Figure 6). Intense cellular and matrix staining for alkaline phosphatase and collagen type I was observed throughout the defect region in all animals where L-pNIPAM-co-DMAc had been injected, regardless of whether HAPna and/or MSCs were incorporated (Figure 6). Immunopositive matrix staining for collagen type I was observed in the same position as the collagen staining observed via Masson's trichrome. Collagen type X, was also expressed within the defect region of all animals, however increased immunopositivity were identified histologically in sham operated controls in comparison to L-pNIPAM-co-DMAc injected animals (Figure 6). High levels of immunopositivity for osteopontin and osteocalcin were observed in 2/6 sham operated controls where effective healing was evident; however in the 4 remaining sham operated controls and L-pNIPAM-co-DMAc injected animals, low levels of cellular staining for both late phase bone markers were observed (Figure 6).

#### FTIR analysis of femur defect region in female exbreeder rats

FTIR imaging was performed to determine the location of the L-pNIPAM-co-DMAc within each bone section and was achieved by plotting the integrated intensity of infrared peaks specific to the different components within the sample. The amide I peak (~1660 cm^−1^) was used to elucidate the location of the bone tissue, but no attempt was made to distinguish between the different bone matrix species. The peak associated with the carbonyl of the DMAc comonomer was used to show the prevalence of the hydrogel and the C-H bending mode ~1460 cm^−1^ was used to determine the distribution of the embedding wax (Supplementary Figure 3).

**Figure 7:**
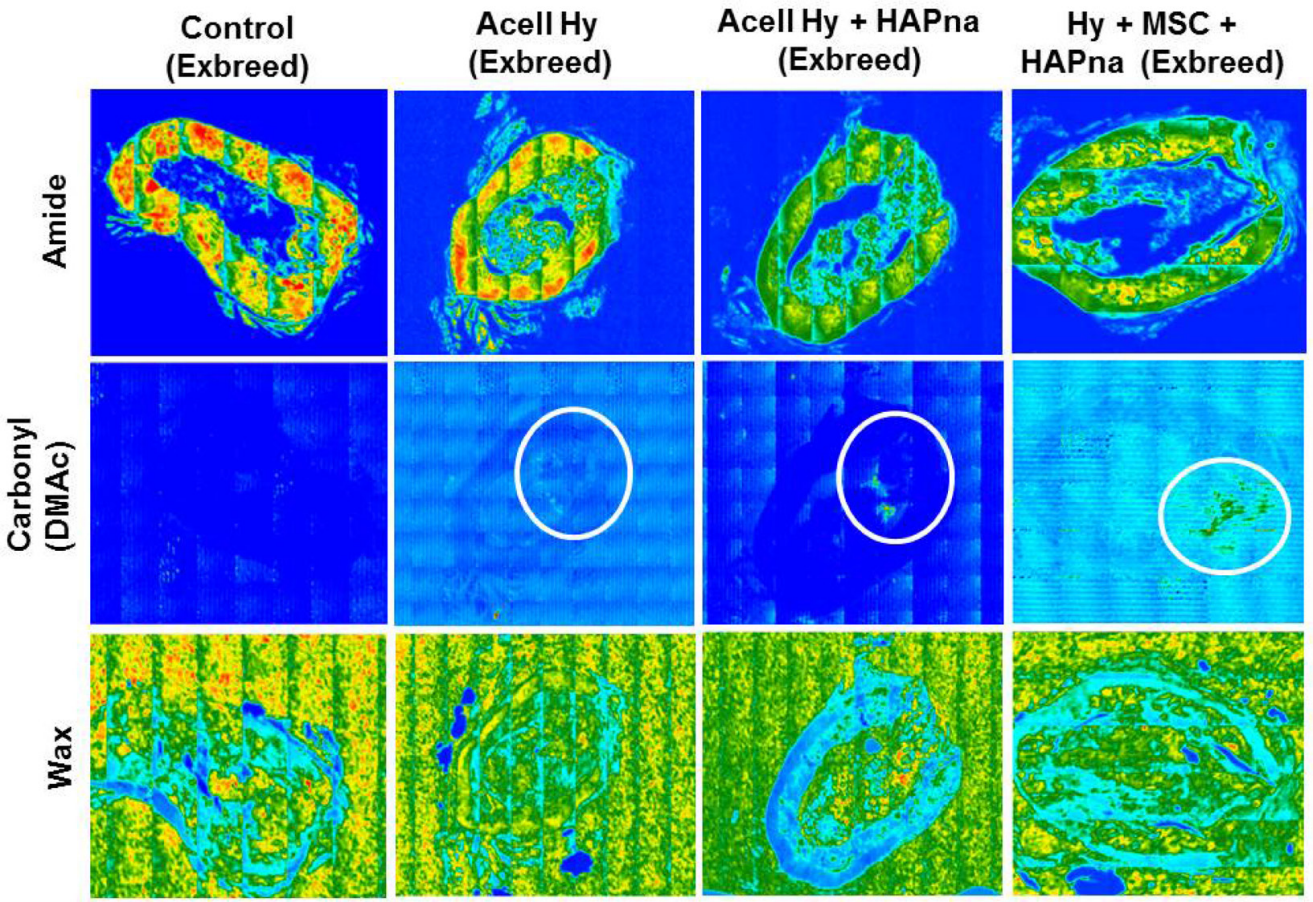
Representative FTIR tissue distribution map of a transverse section within the defect region of paraffin embedded rat femur Overlapping regions displayed between bone tissue (Amide group- 1661 cm^−1^) and the pNIPAM-co-DMAc hydrogel (identified via the carbonyl group on DMAc 1738 cm^−1^). Distribution map of wax (1483 cm^−1^) also included as a control.

There was no evidence of any hydrogel in the control sample (Figure 7). Images generated for the samples that were injected with L-pNIPAM-co-DMAc, with or without HAPna and/or MSCs, showed co-localisation of the hydrogel and bone tissue within the defect zone indicating good integration with the newly formed bone tissue (Figure 7).

## DISCUSSION

Tissue engineering strategies which combine matrix secreting cells with a functional support scaffold, offer a promising alternative to autologous bone grafts for repair of bone defects. The aim of this study was to evaluate the safety and efficacy of L-pNIPAM-co-DMAc containing HAPna and MSCs, within a rat non-critical sized femur defect model following 4 weeks repair time. The femur defect model used in this study is a self-healing defect and therefore enables the evaluation of whether the hydrogel could promote and accelerate bone repair, in comparison to the native animal healing response. We evaluated several clinical requirements within the femur defect model, including: biocompatibility; injection of the hydrogel; maintenance of the hydrogel within the defect region; integration with surrounding bone tissue as well as the osteogenic potential to differentiate MSCs into bone matrix secreting cells for the regeneration and augmentation of bone tissue.

### L-pNIPAM-co-DMAc biocompatiblity and delivery

The clinical translation of suitable bone graft materials is reliant on excellent biocompatibility [38, 39]. The pre-set hydrogel constructs subcutaneously implanted in this study were shown to be biocompatible, with no evidence of a local or systemic inflammatory response, or organ toxicity. Hydrogel constructs were shown to be biocompatible regardless of whether the copolymer, DMAc, was incorporated to tailor the gelation temperature of the hydrogel to 37° C. Furthermore, reduction of the polymerisation time from 24 h to 4 h, reducing the overall synthesis duration, did not affect biocompatibility. In addition, no evidence of toxicity or inflammation was observed where L-pNIPAM-co-DMAc, with and without incorporated HAPna and MSCs, were injected as a liquid into the femur defect, before *in situ* gelation. Many hydrogel systems, previously reported, have not been fully reacted prior to injection, requiring co-injection with crosslinking agents [40–42]; this raises significant safety concerns to surrounding tissues during hydrogel delivery and precludes the incorporation of regenerative cells. The biocompatibility of L-pNIPAM-co-DMAc in the liquid and gelled state, demonstrated here *in vivo,* is extremely promising for patient administration as it provides the capacity for cell delivery and minimally invasive injection. This reduces operative risk and minimises the surgical disturbance of surrounding tissues associated with the implantation of bone graft materials [43, 44]. Gelation of L-pNIPAM-co-DMAc at physiological temperature, within the bone defect, is advantageous over numerous photo-crosslinkable hydrogels developed for bone repair. As the use of UV light to induce *in situ* gelation poses safety concerns to both delivered cells and surrounding tissues during administration [45, 46].

One of the major concerns of injectable biomaterials, particularly with incorporated stem cells with potency to differentiate into multiple cell types, is controlling the location of biomaterial and delivered cells following injection [46, 47]. All L-pNIPAM-co-DMAc hydrogels, demonstrated rapid gelation (<5 s) and were still maintained within the bone defect in all animals after 4 weeks. However, newly regenerated bone matrix extended past the bone cortex region and within the bone marrow space where the hydrogel had been injected. It is essential that hydrogels delivered as liquids are injected with precision within the defect location to prevent spillage and leakage to surrounding tissues [48, 49]; however within the femur defect model, the bone injury spans the entire thickness of the cortex and leaves the bone marrow cavity space exposed. It is likely that L-pNIPAM-co-DMAc will be most suitable for applications to fill bone defects in clinical cases of osteoporosis, smaller bone fractures where irregular shaped defects are contained within intact bone, within spinal fusions where spinal cages are used providing a contained space and periodontal regenerative therapy [43, 50].

### Efficacy of L-pNIPAM-co-DMAc for bone augmentation

The ability of L-pNIPAM-co-DMAc, **±** HAPna and MSCs, to promote and accelerate bone healing within a self-healing femur defect model, was evaluated to determine clinical efficacy for bone repair. Successful bone regeneration is reliant on the presence of bone matrix secreting cells, either differentiated osteoblasts or MSCs which have the ability to differentiate into osteoblasts dependant on their surrounding environmental cues. It is therefore critical that the hydrogel is able to act as an osteoinductive extracellular matrix support which facilitates the adhesion and infiltration of these cells. Unlike previous materials where cell penetration has been an issue [51], native osteoblast cells were observed histologically within the centre of the acellular L-pNIPAM-co-DMAc **±** HAPna, in all animals where it had been injected. This osteoblast cell infiltration is essential not only for deposition of newly regenerated bone matrix, but also to support graft integration with adjacent bone tissue. Biomaterial osteointegration is essential to prevent graft extrusion and provide a functional bone/scaffold matrix with optimum mechanical performance [52, 53]. Unlike previous polymeric bone implants which became encapsulated by fibrous tissue following implantation [54], L-pNIPAM-co-DMAc was found to integrate with surrounding bone tissue in all animals, regardless of whether HAPna or MSCs were incorporated.

In young male rats, where L-pNIPAM-co-DMAc was injected, defect repair was less effective where MSCs had been injected. MSC delivery, to induce regeneration of bone defects, has been investigated *in vivo* in many bone defect models, with positive results [55, 56]. The less effective healing response observed in young male rats where MSCs were injected could be due to the *ex vivo* culture and expansion of MSCs performed in this study prior to implantation. This may have resulted in an altered MSC cell phenotype, similar to the reduced osteogenic differentiation capacity and bone forming ability from MSCs cultured *ex vivo* prior to transplantation reported in other studies [57, 58]. It is also possible that delivered MSCs failed to survive since expanded MSCs have been shown *in vivo* to lose their immunosuppressive activity following implantation and subsequently be destroyed [38, 59]. It is likely that the MSCs transplanted within young male rats in this study were not required to accelerate native bone healing since the femur defect model spans the entire bone cortex, exposing the bone marrow space where there is a large population of native MSCs to aid in bone matrix repair. The transplanted MSCs utilised in this study were not autologous to the animal recipient and therefore would have needed to adapt and survive within the defect environment. Only then can they differentiate into osteoblasts, depositing newly regenerated bone matrix and this may have resulted in a delayed healing response. However, in a non-union defect or in individuals whom have a defective bone healing response, due to either age or disease, implanted MSCs may still be required to promote the initial stages of bone repair.

To better reflect the aged target population typically afflicted by impaired bone healing, the femur defect was performed within aged (>6month) female ex-breeder rats. Bone healing of the femur defect in aged female ex-breeder rats was confirmed to be less effective in comparison to young male rats, with a significantly lower % bv within the defect region, following 4 weeks repair time in sham operated controls. This is in agreement with the delayed bone healing in both aged [60] and female [61] rats previously reported [3].

Regeneration of mature mineralised bone tissue is reliant on the orchestrated production and deposition of several matrix proteins which become organised into a unique anisotropic hierarchical structure [62]. Bone tissue formation is initiated by differentiation of MSCs to osteoblasts that synthesise and deposit collagen type I, the main extracellular matrix component of bone tissue [63]. Histologically, enhanced matrix deposition and healing was found within hydrogel injected defects in comparison to sham operated controls with increased deposition of the osteoblast specific transcription factor runx2, as well as the early bone markers alkaline phosphatase and collagen type I. In contrast only low levels of expression for the late bone markers osteopontin and osteocalcin, were observed. This suggests that the matrix repair that had taken place was early on in the bone regeneration process. All L-pNIPAM-co-DMAc formulations were shown to increase deposition of collagen staining which could indicate promotion of initial stages of bone repair, however 4 weeks post operation was not long enough for the production of a fully mature mineralised bone matrix. The impaired healing capacity of aged ex-breeder rats [60], as well as potentially a reduced regenerative capacity of native MSCs and delivered MSCs [64–67], extracted from these animals, is likely to be the reason that complete repair of the defect site was not seen by 4 weeks with no significant difference observed in the bone volume fraction on Micro-CT analysis.

Bone healing in general is initiated at the peripheral margins of bone defects [63], as was observed within sham operated controls. However, where L-pNIPAM-co-DMAC was injected, the mechanism of bone repair appeared to be different, with newly regenerated bone observed in direct association with the hydrogel in the centre of the femur defect. This alternative repair may be clinically beneficial in patients with impaired healing, where bone regeneration initiated at the peripheral margins is insufficient and delayed.

The healing response within the femur defect region between the different hydrogel injected groups was histologically indistinct following 4 weeks repair time. Recently Hayashi *et al.,* 2016 reported that *in vivo* bone repair, using a nanofiber hydrogel, was reliant on the incorporation of stem cells. In this study, bone repair was initiated in the centre of the femur defect even where acellular L-pNIPAM-co-DMAC **±** HAPna was injected and migration of native cells was observed. This suggests that the hydrogel could offer clinical benefit even in the absence of cells [67]. However increased runx2 immunopostivity was observed where MSCs were incorporated within L-pNIPAM-co-DMAC with HAPna, suggesting improved osteogenicity with the delivery of MSCs, in agreement with previous studies [35, 68]. Effective bone repair and scaffold integration was evident where L-pNIPAM-co-DMAc without HAPna had been injected, indicating that the addition of HAPna is not essential within L-pNIPAM-co-DMAc for bone augmentation. This is likely due to the fact that the hydrogel was injected within a bone defect location and was exposed to the native osteogenic biological cues and mechanical forces required to stimulate bone healing. However where L-pNIPAM-co-DMAc was injected without HAPna, hydrogel which was not associated with bone remodelling was also located on the outside of the defect region, suggesting local signalling drives osteogenesis only in close proximity to the bone. Moreover, we have also previously demonstrated that the addition of HAPna within L-pNIPAM-co-DMAc is critical to ensure correct osteogenic cell differentiation *in vitro* [18, 37, 53].

### Considerations for the clinical translation of L-pNIPAM-co-DMAc for bone repair

One of the major concerns with the use of hydrogels for bone repair is their associated weak mechanical properties [39]. In the present study, L-pNIPAM-co-DMAc was mechanically sufficient to provide adequate support for continued animal activity following the femur defect. However, the femur defect was not of a critical size and therefore the load bearing forces applied to the scaffold do not reflect those that the scaffold would need to withstand within a large critically sized bone defect. In general for larger bone defects the use of L-pNIPAM-co-DMAc is likely to be mechanically insufficient in early repair stages, but may be applied as an osteogenic cell delivery vehicle, in combination with an additional support structure to stimulate bone regeneration and promote graft integration. Within the femur defect performed in this study, L-pNIPAM-co-DMAc provided initial support as a scaffold between the adjacent bone surfaces, facilitated native cell infiltration and graft integration. Together with stimulating osteogenic differentiation of native osteoprogenitor cells and transplanted stem cells. This is shown by the production of early osteogenic markers, to promote effective bone augmentation. Therefore in non-weight bearing situations L-pNIPAM-co-DMAc may provide the beneficial properties of both a cell carrier and a support scaffold to regenerate and augment bone in clinical situations such as periodontal disease [69], increasing bone density in osteoporosis [28], aiding fixation of prosthetic implants [70] as well as non-union fracture [71], where mechanical support is provided by an additional implant.

A design consideration for the development of injectable hydrogels for bone repair, which is often regarded as fundamental, is that the developed hydrogel must degrade in a controlled manner to enable effective load transfer to the newly regenerated bone matrix [71]. Scaffold degradation is dependent on the chemical and structural properties of the biomaterial, as well as several biological factors including mechanical loading, pH, temperature, enzymatic activity and rates of tissue ingrowth [20, 72]. Therefore scaffold degradation will inevitably be patient dependant and therefore designing a hydrogel that degrades commensurate with bone in-growth, remains a significant challenge and a barrier to the clinical translation of these treatments. In addition, scaffold degradation raises safety issues regarding the toxicity of degradation products which may cause tissue dysfunction and/or initiate an inflammatory reaction [71]. In the present study L-pNIPAM-co-DMAC subcutaneously implanted for 6 weeks and injected within a femur defect for 4 weeks, demonstrated no signs of scaffold degradation and hence may avoid some of the issues and complications associated with scaffold degradation. Long term *in vivo* studies within a critically sized bone defect are essential to determine the long term fate and efficacy of L-pNIPAM-co-DMAc. However, it is envisaged that the non-biodegradable hydrogel, will facilitate long term scaffold support, whilst the newly regenerated bone tissue, will integrate with the hydrogel and undergo natural bone tissue remodelling, to regenerate a fully integrated and functional bone/hydrogel scaffold matrix.

## CONCLUSION

The current study used an injectable L-pNIPAM-co-DMAc loaded with HAPna and MSCs to induce bone healing within a rat femur defect. We have demonstrated that the injectable hydrogel is biocompatible, able to integrate with surrounding bone tissue and promote increased deposition of early markers of bone formation. The low viscosity nature of L-pNIPAM-co-DMAc, enables its delivery directly into the target site, where it can fill both micro and macro fractures. Providing a scaffold between adjacent surfaces of bone tissue and initial support, facilitate the migration of native cells to aid tissue integration as well as promote the osteogenic differentiation of transplanted stem cells to regenerate and augment a functionally integrated bone matrix. This system could potentially provide safe and efficacious bone regeneration for the treatment of small bone defects in non-union cases as well as clinical cases of osteoporosis, fixation of prosthetic implants, spinal fusion and periodontal regenerative therapy.

## MATERIALS AND METHODS

### Hydrogel synthesis

#### Synthesis of L-pNIPAM hydrogel

An exfoliated suspension of Laponite^®^ clay nanoparticles (25–30 nm diameter, <1 nm thickness) (BYK Additives Ltd, Cheshire UK) was prepared by vigorous stirring of Laponite^®^ (0.1 g) in deionised H_2_0 (18 MΩ) (9.0 mL) for 24 h. N-isopropylacrylamide 99% (NIPAM) (0.9 g) (Sigma, Poole, UK) and 2–2ʹ-azobisisobutyronitrile (AIBN) (9 mg) (Sigma, Poole, UK) were added to the suspension and stirred for 1 h. After passing the suspension through 5–8 μm pore filter paper, polymerisation was initiated by heating to 80° C and the reagents were allowed to react for either 4 h (L-pNIPAM-4h) or 24 h (L-pNIPAM-24h), to investigate the effect of reduced synthesis time on the biocompatibility of pre-set hydrogel constructs following subcutaneous implantation (see section 2.2.1). It was observed that after heating the monomeric suspension to 80° C, the transparent liquid transformed to a milky suspension. Following 4 (L-pNIPAM-4h) or 24 h (L-pNIPAM-24h) the hydrogel suspension was cooled to room temperature which resulted in a solidified hydrogel.

#### Synthesis of L-pNIPAM-co-DMAc with or without hydroxyapatite nanoparticles

Synthesis of L-pNIPAM-co-DMAc was performed as described previously [18] polymerisation at 80° C was performed for 4 h (L-pNIPAM-co-DMAc-4h) or 24 h (L-pNIPAM-co-DMAc-24h). For hydroxyapatite containing hydrogels the L-pNIPAM-co-DMAc was cooled to 50° C and hydroxyapatite nanoparticles (HAPna) (<200 nm) (Sigma, Poole UK) were homogenously mixed into the liquid hydrogel suspension at 0.5 mg/mL as describe previously [18].

### Animals and housing

Animal experiments were performed under Project Licences PPL 40/3311and PPL 708054 issued by the Home Office under the Animals (Scientific Procedures) Act of 1986. Individual protocols and study plans were approved by the local Animal Welfare and Ethical Review Body. A total of 83 (51 male and 32 female) Wistar rats were used in this study. Male rats were supplied by Harlan UK and were approximately 12 weeks old at surgery with an average weight of 327 g for the subcutaneous model and 385 g for the femur model. Female rats used in this study were older (>6 months) ex-breeders, obtained from (Charles Rivers, Harlow, UK) with an average body weight of 411 g at surgery. All animals were of a healthy status and were genetically unmodified. All animals were housed in groups and kept in standard laboratory conditions with free access to food (Teklad Global 18% Protein Rodent Diet, Harlan Laboratories, UK) and water.

### *In vivo* safety: subcutaneous implantation

#### Subcutaneous implantation operational procedure

Initial biocompatibility of the hydrogel was evaluated by subcutaneous implantation of hydrogel constructs in young 10–12 weeks old male Wistar rats for 6 weeks. Four separate hydrogel batches were investigated: L-pNIPAM-4h; L-pNIPAM-24h; L-pNIPAM-co-DMAc-4h and L-pNIPAM-co-DMAc-24h together with sham operated controls (*n* = 6). Reduced preparation time (4 or 24 h) was investigated to determine whether hydrogel synthesis could be optimised for future in-clinic use. Hydrogels for implantation were prepared by pipetting 300 μl of liquid L-pNIPAM or L-pNIPAM-co-DMAc onto sterile petri dishes at RT, resulting in solidified cylindrical hydrogel discs (2mm height, 14mm diameter). All hydrogel scaffolds were sterilised prior to surgical implantation by UV radiation (230V, 50/60Hz, 0.400KW) for 15 minutes using TelSTAR mini-V/PCR laminar flow cabinet with built in UV light.

Thirty 12-week old male Wistar rats with an average weight of 327 g were used for this investigation. Anaesthesia was induced using 5% Isofluorane/O_2_ (Abbott Laboratories, Maidenhead, UK) and maintained with 1.5 – 2% Isofluorane/O_2_. Surgical site was prepared by swabbing with Tisept^®^ (Medlock Medical Ltd, Oldham UK). A midline incision was made through the skin on the back and a pocket developed on the left side by blunt dissection. One hydrogel disc was placed in the pocket and the wound closed with Vicryl^™^ sutures (Ethicon^™^, Johnson and Johnson, Livingston, Scotland). Sham operated control animals were treated in an identical manner but no implant placed. After 6 weeks rats were sacrificed using schedule one method and weights recorded. Implantation site and organs including: liver; kidney; testes and lymph nodes were removed and fixed in 10% w/v neutral buffered formalin (nbf) (Leica Microsystems, Milton Keynes UK) for histological assessment.

#### Blood collection and sample analysis

To investigate evidence of systemic inflammatory response or organ pathology, a complete blood count, differential blood count and serum biochemistry analysis was performed. At sacrifice, ~1.5mL of blood was obtained via cardiac puncture and collected into three vials: ~1.0mL of blood was collected into EDTA vials for full blood count and differential blood count, 150μL of blood was collected into a serum coagulation vial for serum biochemistry analysis and a further 50 μL was collected into a second serum coagulation vial for assessment of c-reactive protein (CRP) (IDEXX laboratories, Ludwigsburg, Germany). Haematological parameters analysed as part of the full blood count and differential blood count included: haemoglobin (g/dL), packed cell volume (%), mean cell volume (fL), mean corpuscular height (pg), mean corpuscular haemoglobin concentration (g/dL), erythrocytes (T/L), leukocytes (g/L), lymphocytes (absolute/μL), thrombocytes (g/dL), eosinophils (absolute/ μL), band neutrophils (absolute/μL), segmented neutrophils (absolute/μL), and monocytes (absolute/μL). A differential white blood cell count was also assessed morphologically by blood smears. Serum biochemistry parameters investigated included: alkaline phosphatase (U/L), albumin (g/L), alanine transferase (U/L) and bilirubin (μmol/L) as a measure of liver function, as well as creatinine (μmol/L) as a measure of kidney function. Samples taken for full blood count and differential blood count were shipped at ambient temperature and samples for serum biochemistry and CRP analysis were shipped on dry ice. All samples were analysed independently by IDEXX laboratories (Ludwigsburg, Germany).

### *In vivo* efficacy rat femur defect model

#### Experimental design: rat femur defect model in 10–12 weeks old male Wistar rats

For assessment of hydrogel biocompatibility and performance in bone, a healing femur defect model was used. A 1mm defect was created in the mid-shaft of the right femur of 10–12 week old male Wistar rats (average weight 385 g). Animals were divided into four separate experimental groups to investigate the efficacy of the L-pNIPAM-co-DMAc ± MSCs and HAPna for bone repair. The experimental groups were divided as follows: Sham operated control; L-pNIPAM-co-DMAc + 2 × 10^6^ cells/mL rat MSCs; acellular L-pNIPAM-co-DMAc + 0.5 mg/mL HAPna; or L-pNIPAM-co-DMAc + 2 × 10^6^ cells/mL rat MSCs + 0.5 mg/mL HAPna (*n* = 3 per group).

#### Experimental design: rat femur defect model in aged (>6 months) female ex-breeder Wistar rats

Femur defects were created in female ex-breeder Wistar rats (>6 months, average weight at surgery of 411 g) with the hypothesis that these animals would have a reduced or delayed healing capacity which represents the aged population which would be targeted for bone repair. To test this hypothesis initial experiments investigated the healing of sham operated controls in 10–12 week old male Wistar rats in comparison to the older (>6 months) female ex-breeder Wistar rats (*n* = 7). Following this, further animal surgeries were performed to investigate the efficacy of the L-pNIPAM-co DMAc ± MSCs and HAPna for bone repair in aged female ex-breeder Wistar rats. The experimental groups were: acellular L-pNIPAM-co-DMAc ± 0.5 mg/mL HAPna; L-pNIPAM-co-DMAc + 2 × 10^6^ cells/mL rat MSCs + 0.5 mg/mL HAPna (*n* = 7 for each group). All animals were maintained for 4 weeks prior to sacrifice for analysis.

#### Extraction of rat mesenchymal stem cells

Rat MSCs were isolated from the same breeding line of male 10–12 week old male Wistar rats (pooled from *n* = 5) and older (<6 month) female ex-breeder Wistar rats (pooled from *n* = 4). Animals were sacrificed using schedule 1 killing. Tibiae and femora were aseptically removed, bones dissected to expose marrow space and bone marrow flushed out using 18 gauge needle with DMEM (Life Technologies, Paisley UK) supplemented with 10% v/v heat inactivated foetal calf serum (FCS) (Life Technologies, Paisley UK), 100 U/ml penicillin (Life Technologies Paisley UK), 100 μg/ml streptomycin (Life Technologies Paisley UK), 250 ng/ml amphotericin (Sigma, Poole UK), 2 mM glutamine (Life Technologies, Paisley UK) and 10 μg/ml ascorbic acid (Sigma, Poole UK) (complete cell culture media). Mononuclear cells were then isolated as previously described [73] using a Histopaque 1077 gradient (Sigma, Poole, UK). MSCs (characterised by their adherence to plastic and morphology) were then expanded in monolayer and used for implantation at passage 2.

#### *In vivo* efficacy rat femur defect operational procedure

Anaesthesia was induced and maintained as described above. A single dose of 0.05ml of 50mg/ml carprofen (Rimadyl^™^, Pfizer Ltd, Sandwich UK) was given by subcutaneous injection. The right hind limb was immobilised and the surgical site prepared by swabbing with Tisept^®^. An incision was made over the right femur and bone exposed by blunt dissection; a single defect was created using a 1mm diameter stainless steel bur with saline irrigation. The defect was either left untreated to serve as a sham operated control or injected with L-pNIPAM-co-DMAc as described previously (section 2.2.2.1). Surgical wounds were closed with resorbable sutures. Cell seeding solutions were prepared at a density of 2 × 10^6^ cells/mL, homogenously mixed with the L-pNIPAM-co-DMAc suspension (38–39° C) ± 0.5 mg/mL HAPna (Sigma, Poole, UK) and injected directly into the femur defect via 26-gauge needle injection (Becton Dickinson, Plymouth, UK) until the defect cavity was full with the implanted hydrogel. Following injection, it was observed that the hydrogel solidified rapidly (<5 seconds) within the bone defect site.

### Microcomputed tomography

Microcomputed Tomography (Micro-CT) was performed 4 weeks after surgery on the defect site in the femur using a Bruker SkyScan 1172, images were reconstructed with NRecon v1.6.10.4 software and analysis performed using CTAn v1.4.0. μCT parameters were: X-ray source, 65kV/139uA; rotation 360°; exposure time 1180 ms; image pixel size 10 μm; 0.5 μm aluminium filter. A cylindrical region of interest (surrounding the femur defect) was selected with a diameter of 1mm and depth of 1mm, which included the entire defect region within the mid-shaft of the femur. Parameters for image processing were: ring artefact correction: 10; beam hardening correction: 15%; global thresholding: 80–225. 3D images of the defect region and new bone formation, using double-time cubes algorithm, were reconstructed for representation and the bone volume (%) was measured.

### Histological assessment of subcutaneous implantation site, femur defect site and organs

For histological assessment of hydrogel biocompatibility, tissue integration and bone augmentation, the implant sites (subcutaneous implant skin region and femur defect region) together with vital organs were extracted and fixed in 10% w/v formalin (Leica Microsystems, Milton Keynes UK) overnight (organs) or for 1 week (skin region and femur specimens). The femur specimens were then decalcified for 3 weeks in Surgipath Decalcifier I (Leica Microsystems, Milton Keynes UK), with solution changed every 2 days, prior to routine paraffin embedding. Tissue samples were serially sectioned at 4μm and sections mounted every 100μm onto positively charged slides (Leica Microsystems, Milton Keynes UK) for histological evaluation throughout the defect region. Sections stained with haematoxylin and eosin (H&E) Masson's trichrome as described previously [18]. All slides were blinded and independently assessed by a histopathologist.

### Immunohistochemistry assessment of femur defect site

Immunohistochemistry protein expression of the macrophage marker CD68, the early bone markers runx2 and alkaline phosphatase, the bone matrix markers collagen type I and collagen type X, as well as the late bone markers osteopontin and osteocalcin were selected for immunohistochemistry (IHC) investigation to assess the bone healing response within the rat femur defect model following 4 weeks repair time. Sections were then prepared as described above; IHC was performed as previously described (Table 1) [18].

**Table 1:** Target antibodies used for IHC, their optimal concentrations and antigen retrieval methods [18]

Target Antibody	Clonality	Optimal Dilution	Antigen Retrieval	Secondary Antibody	Serum Block
CD68	Mouse Monoclonal	1:200	Enzyme	Rabbit anti mouse	Rabbit
Runx2	Mouse Monoclonal	1:200	Heat	Rabbit anti mouse	Rabbit
Alkaline Phosphatase	Rabbit Polyclonal	1:200	Heat	Goat anti rabbit	Goat
Collagen type I	Rabbit Polyclonal	1:200	Enzyme	Goat anti rabbit	Goat
Collagen type X	Rabbit Polyclonal	1:400	Enzyme	Goat anti rabbit	Goat
Osteopontin	Mouse Monoclonal	1:200	None	Rabbit anti mouse	Rabbit
Osteocalcin	Mouse Monoclonal	1:400	Enzyme	Rabbit anti mouse	Rabbit

### Fourier transform infrared spectroscopy image analysis of femur defect sections

Femur specimens were processed and sectioned as described above (section 2.5). For FTIR image analysis a 4 μm transverse section within the centre of the femur defect region was mounted onto gold coated reflective slides (Kevley Technologies, Ohio, USA). Mid-infrared microscopic images were collected using an Agilent 680-IR FTIR spectrometer coupled with a FTIR imaging microscope. The microscope was fitted with a liquid nitrogen cooled 128 × 128 mercury-cadmium-telluride focal plane array detector (FPA) and an automated sampling stage. Images were collected and processed using Resolutions Pro FTIR Spectroscopy software version 5.2.0 (Agilent Technologies, UK). FTIR mosaic images encompassing the whole of the bone sections were collected in transflectance mode by co-adding 128 scans at a spectral resolution of 8 cm^−1^ using a clean region of the reflective substrate as a background. Species specific images were generated by integrating the peaks ~1738, ~1660 and ~1460 cm^−1^, to obtain the distribution of L-pNIPAM-co-DMAc, bone tissue and embedding wax respectively within each section.

### Data analysis, processing and statistical analysis

All slides were examined with an Olympus BX51 microscope and images captured by digital camera and Capture Pro OEM v8.0 software (Media Cybernetics, Buckinghamshire, UK). Histological sections were analysed, features noted and images captured to document their histological appearance. The percentage area of collagen staining was assessed on slides stained with Masson's trichrome using image J analysis software version 1.5i. The entire defect region (1mm diameter, 1mm depth) was selected as the region of interest. The image was split into red, green and blue using RGB stacks and thresholding was applied at a range of 10–120 using the red channel which gave the best contrast for blue (collagen) staining; the percentage area of blue staining was then measured.

Evaluation of IHC staining was performed by counting immuno positive and immuno negative cells for each section and immunopositive cells expressed as a percentage of total count.

All tests were performed at least in triplicate. Data were assessed for normality using the Shapiro Wilks test and found to be non-normally distributed, as such data were non-parametric and hence statistical comparisons were performed by Kruskal-Wallis for pairwise comparisons (Conover-Inman) with statistical significance accepted at *p* ≤ 0.05. Pairwise comparisons were made as follows: between all implant conditions for blood and biochemistry analysis (Figure 1C), between all experimental groups within young 10–12 weeks old male rats for micro CT analysis, between all experimental groups within aged exbreeder female rats for Micro-CT analysis (Figure 4) and between all experimental groups within aged exbreeder female rats for % area of collagen staining (Figure 5). Statistical analysis of the two groups: sham operated young male rats vs sham operated aged exbreeder female rats for micro CT analysis was performed by Mann–Whitney *U* test. Data were then presented on graphs; all replicates have been shown with median value indicated to demonstrate clearly the spread of replicates.

## SUPPLEMENTARY MATERIALS FIGURES AND TABLES





## Abbreviations

L-pNIPAM-co-DMAc: Laponite^®^ crosslinked pNIPAM-co-DMAc hydrogel
HAPna: Hydroxyapatite nanoparticles
MSCs: Mesenchymal stem cells
CRP: c-reactive protein
FCS: Foetal calf serum
Micro-CT: Microcomputed tomography
H&E: haematoxylin and eosin
IHC: immunohistochemistry
Runx2: Runt related box 2
FTIR: Fourier-transform infrared spectroscopy
FPA: Focal plane array detector
% bv: Percentage bone volume.
