# Supplementary material for: In vivo safety and efficacy testing of a thermally triggered injectable hydrogel scaffold for bone regeneration and augmentation in a rat model
Source: Oncotarget. 2018 Apr 6;9(26):18277–95. doi: 10.18632/oncotarget.24813 (PMC5915072; doi:10.18632/oncotarget.24813)
Supplement: Supplementary file 1 [file oncotarget-09-18277-s001.pdf]

In vivo safety and efficacy testing of a thermally triggered injectable hydrogel scaffold for bone regeneration and augmentation in a rat model

SUPPLEMENTARY MATERIALS

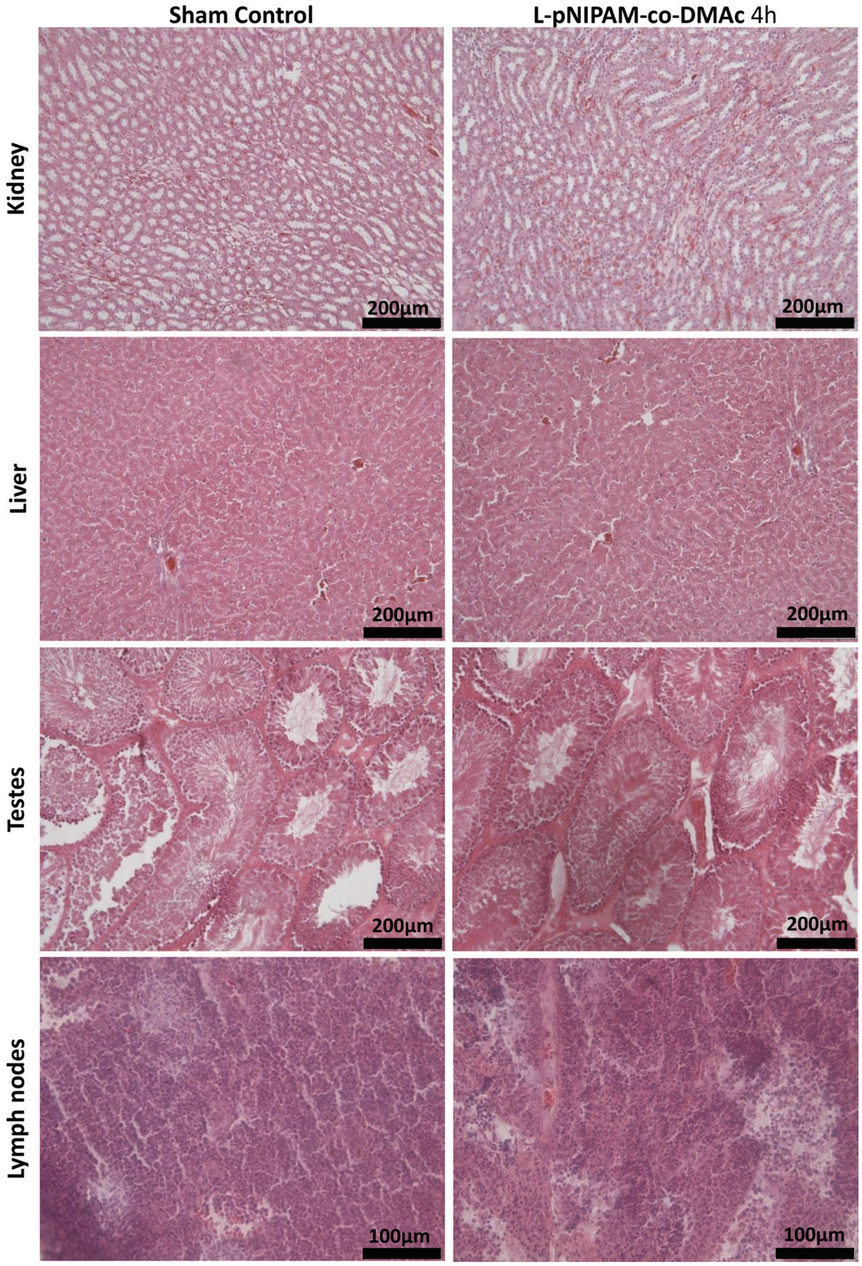

Supplementary Figure 1: Initial biocompatibility of the hydrogel was evaluated by subcutaneous implantation of hydrogel constructs in young 10–12 weeks old male Wistar rats for 6 weeks. Representative microscopic mages of kidney, liver, testes and lymph nodes stained with H&E from either sham operated control animals or hydrogel (L-pNIPAM-co-DMA-4h) implanted animals. Scale bar: 200 µm or 100 µm.

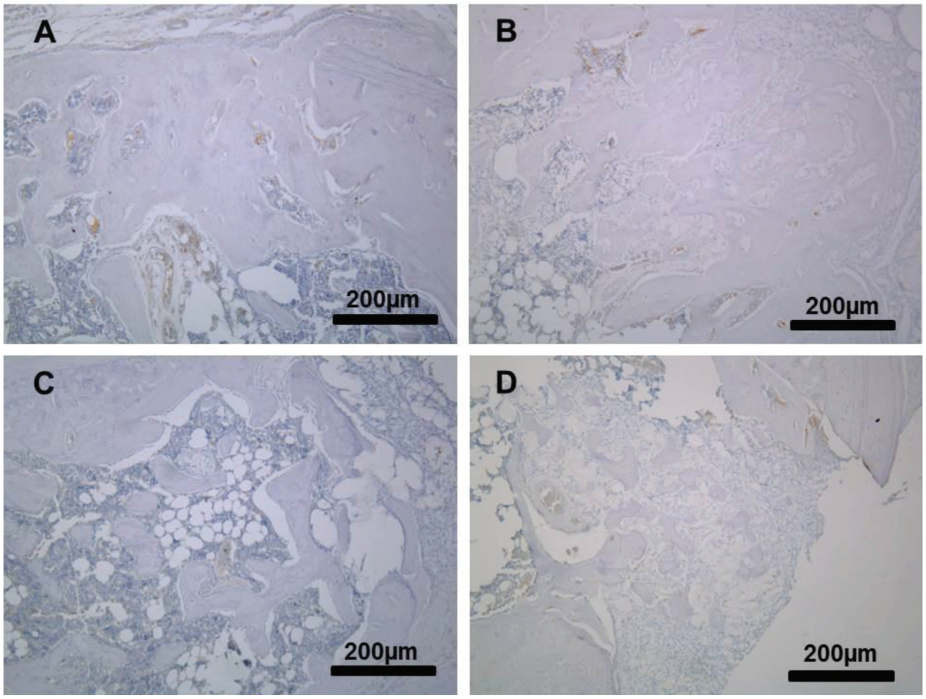

Supplementary Figure 2: CD68 Immunohistochemistry assessment of the defect site from in exbreeder female (>7months old) white wistar rats. A non critical sized defect was created in the midshaft of the femur, left void to serve as a control or injected with with L-pNIPAM-co-DMAc hydrogel with/without MSCs and HAPna and maintained for 4 weeks. Image shown represents an animal selected from each experimental group where the mid-range bone repair was observed from n = 6 replicates. Experimental groups: (**A**) Sham operated controls, (**B**) acellular L-pNIPAM-co-DMAc hydrogel, (**C**) acellular L-pNIPAM-co-DMAc hydrogel with HAPna, and (**D**) L-pNIPAM-co-DMAc hydrogel with incorporated MSCs and HAPna. Scale bar: 200 µm.

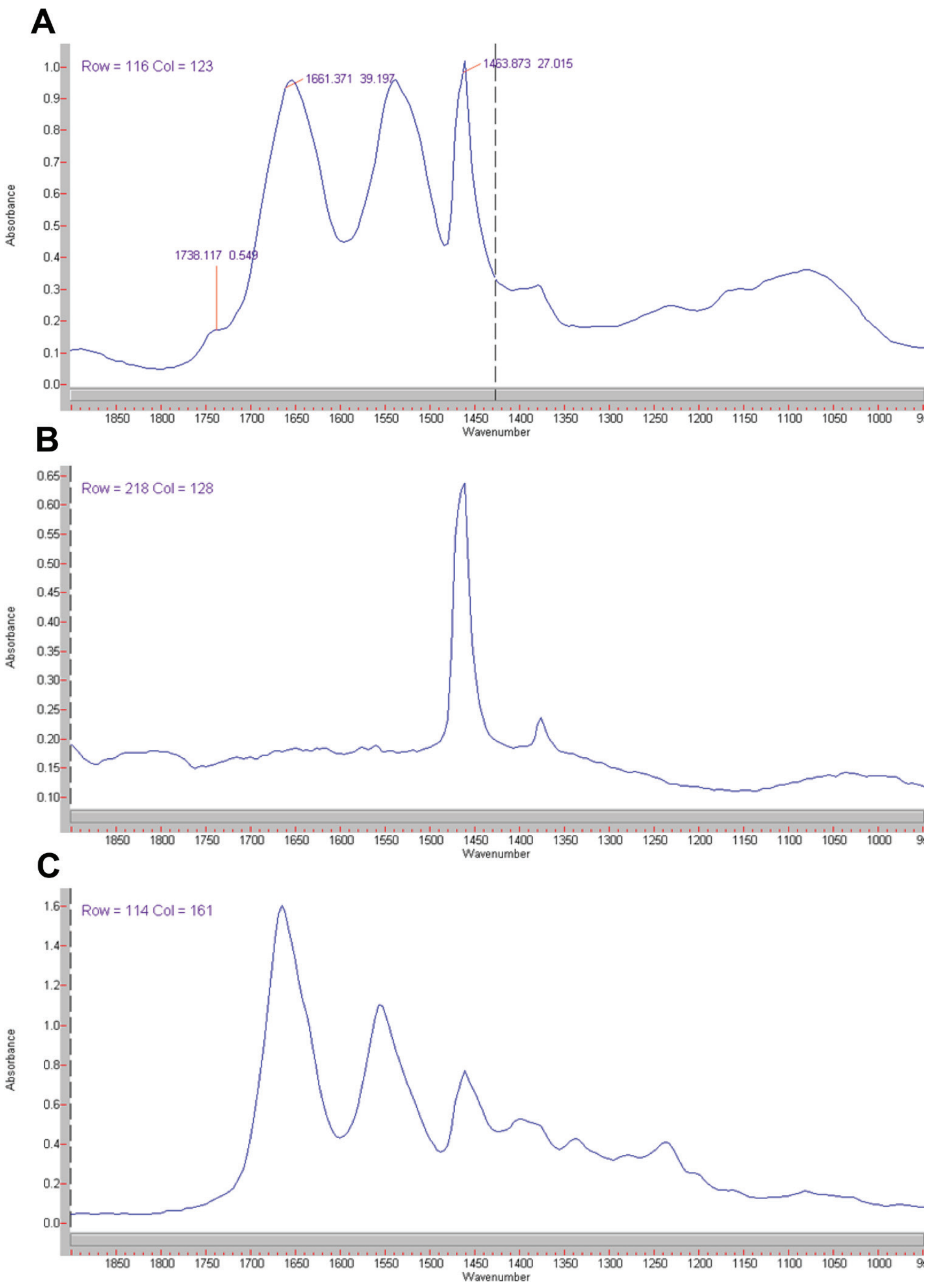

Supplementary Figure 3: Typical FTIR spectrums showing carbonyl (1738), amide (1661) and wax (1483) (**A**), wax (**B**) and tissue region away from defect (**C**).

Supplementary Table 1: Hydrogels with MSCs investigated for bone regeneration. See Supplementary_Table_1

Supplementary Table 2: Alizarin red staining of implantation sites

Animal Replicate	L-pNIPAM-4h	L-pNIPAM-24h	L-pNIPAM-co-DMAc 4h	L-pNIPAM-co-DMAc-24h
1	×			
2				×
3	×	×	×	×
4	×	×		
5		×		
6	×			

(X) Indicates animals where calcium deposits were observed within the hydrogel.
