# Supplementary material for: In vivo safety and efficacy testing of a thermally triggered injectable hydrogel scaffold for bone regeneration and augmentation in a rat model
Source: Oncotarget. 2018 Apr 6;9(26):18277–95. doi: 10.18632/oncotarget.24813 (PMC5915072; doi:10.18632/oncotarget.24813)
Supplement: Supplementary file 2 [file oncotarget-09-18277-s002.docx]

| Hydrogel Scaffold | Culture conditions | Addition of Osteogenic factors? | Cell Type | Key Findings | Ref |
| --- | --- | --- | --- | --- | --- |
| Photocrosslinkable poly (ethylene glycol) PEG hydrogel | *In vitro* | Osteogenic differentiating medium | Human MSC | After 1 week in culture with osteogenic differentiating medium, MSCs encapsulated within PEG hydrogels, differentiated into osteoblasts with expression of ALP, osteopontin and osteonectin with mineralised matrix staining. | [1] |
| Electrospun poly(e-caprolactone) (PCL)  scaffold. | *In vivo* rat omenta implantation | Cells seeded scaffolds were cultured in osteogenic medium for 4 weeks prior to implantation. | Rat MSC | Constructs maintained the size and shape of the original scaffolds after 4 weeks. Mineralization and type I collagen, assessed histologically and by immunohistochemistry, was detected in the MSC scaffolds | [2] |
| Acrylated hyaluronic acid hydrogel | *In vivo* rat calvarial defect. | BMP-2 | Human MSC | Hydrogel with BMP2 and MSCs had the highest osteocalcin expression and mature bone formation in comparison to controls, 4 weeks post-surgery. | [3] |
| Hydroxyapatite (HA) and poly(NiPAAm-co-AAc) hydrogel. | *In vivo* rat subcutaneous model. | BMP-2 | Rabbit MSC | Bone formation was histologically observed with increased ALP and osteocalcin expression throughout the cell seeded scaffolds containing growth factor BMP2. | [4] |
| Polycaprolactone collagen type I hydrogel | *In vitro* | Osteogenic differentiating medium | Human MSC | MSCs encapsulated within collagen hydrogel underwent osteogenic differentiation when cultured under osteogenic differentiating medium, with expression of ALP, collagen type I and osteocalcin. | [5] |
| Collagen type I hydrogel | *In vitro* | Osteogenic differentiating medium | Rat MSC | Collagen type I hydrogels enabled the attachment, migration, proliferation and osteogenic differentiation of rat MSCs, with expression of ALP, when cultured in osteogenic differentiating medium. | [6] |
| Injectable, thermoresponsive hyaluronic acid-chitosan-poly(N-isopropylacryamide) (HA-CPN) copolymer hydrogel. | *In vitro* and *In vivo* subcutaneous nude mice model | In vitro experiments were performed in osteogenic differentiating medium. MSCs were pre differentiated into osteoblasts prior to *in vivo* transplantation. | Canine MSC | Cells proliferated more and expressed higher levels of ALP in HA-CPN hydrogel than on tissue culture plates following osteoinduction. *In vivo* studies demonstrated that the HA-CPN hydrogel was biocompatible and ectopic bone bone formation was observed when the hydrogel with encapsulated osteo-induced MSCs were translated subcutaneously. | [7] |
| Polysaccharide hydrogels compared with commercial calcium-phosphate ceramics investigated alone and in combination. | *In vivo* anterolateral rat cortical bone defect. | n/a standard culture medium | Syngenic rat MSC | Minimal healing was observed in controls with bone repair observed in hydrogel. Newly regenerated bone matrix was obsved in hydrogel (20% BV/TV) and ceramic (26% BV/TV) treated defects; enhanced bone repair was obsrved with delivery of MSCs within hydrogel (>26% BV/TV). | [8] |
| Self-assembling peptide nanofiber hydrogel | *In vivo* rat calvarial defect. | Cells were pre differentiated in osteogenic differentiating medium | iPSC | Minimal healing in defects filled with salt solution (control) and nanofiber hydrogel alone. Increased bone regeneration in defects with iPSC with nanofiber hydrogel. | [9] |
| poly(ethylene glycol)-poly(l-lactic acid) (PEG-PLLA) copolymer hydrogel | In vivo rat femoral bone segmental defect model. Defects were fixed with a polyethylene fixation plate and filled with hydrogel | MSCs were treated with BMP2 or transfected with BMP2 nonviral vector. Some scaffolds were also wrapped with a layer of preharvested periosteum to provide osteogenic factors. | Rat MSCs | The differentiation of the MSCs was not very efficient without the addition of BMP2 or periosteum. The addition of the periosteum with MSCs and the PEG-PLLA scaffolds, promoted bone regeneration. The largest bone volume within the defect region was observed in where BMP2 transfected MSCs were implanted. | [10] |
| Injectable calcium phosphate with alginate hydrogel fibers | *In vitro* | Cells were pre differentiated in osteogenic differentiating medium | Human induced pluripotent stem cell-derived mesenchymal stem cells (hiPSC-MSCs), dental pulp stem cells (hDPSCs) and bone marrow MSCs (hBMSCs) | Injection of the hydrogel and encapsulated cells through a 10 gauge needle did not affect cell viability. All cell types proliferated and differentiated down the osteogenic lineage inside the hydrogel fibers with calcium phosphate, cultured in osteogenic medium. | [11] |

***Supplementary table 1:*** *Hydrogels with MSCs investigated for Bone regeneration.*

**Supplementary table 1 references**

1. Nuttelman CR, Tripodi MC, Anseth KS. In vitro osteogenic differentiation of human mesenchymal stem cells photoencapsulated in PEG hydrogels. Journal of Biomedical Materials Research Part A. 2004; 68:773-782.

2. Shin M, Yoshimoto H, Vacanti JP. In vivo bone tissue engineering using mesenchymal stem cells on a novel electrospun nanofibrous scaffold. Tissue Eng. 2004; 10:33-41.

3. Kim J, Kim IS, Cho TH, Lee KB, Hwang SJ, Tae G, Noh I, Lee SH, Park Y, Sun K. Bone regeneration using hyaluronic acid-based hydrogel with bone morphogenic protein-2 and human mesenchymal stem cells. Biomaterials. 2007; 28:1830-1837.

4. Na K, Sun BK, Woo DG, Yang HN, Chung HM, Park KH. Osteogenic differentiation of rabbit mesenchymal stem cells in thermo-reversible hydrogel constructs containing hydroxyapatite and bone morphogenic protein-2 (BMP-2). Biomaterials. 2007; 28:2631-2637.

5. Reichert J, Heymer A, Berner A, Eulert J, Nöth U. Fabrication of polycaprolactone collagen hydrogel constructs seeded with mesenchymal stem cells for bone regeneration. Biomedical Materials. 2009; 4:065001.

6. Hesse E, Hefferan TE, Tarara JE, Haasper C, Meller R, Krettek C, Lu L, Yaszemski MJ. Collagen type I hydrogel allows migration, proliferation, and osteogenic differentiation of rat bone marrow stromal cells. Journal of Biomedical Materials Research Part A. 2010; 94:442-449.

7. Liao H, Chen C, Chen J. Osteogenic differentiation and ectopic bone formation of canine bone marrow-derived mesenchymal stem cells in injectable thermo-responsive polymer hydrogel. Tissue Engineering Part C: Methods. 2011; 17:1139-1149.

8. Frasca S, Norol F, Le Visage C, Collombet J, Letourneur D, Holy X, Sari Ali E. Calcium-phosphate ceramics and polysaccharide-based hydrogel scaffolds combined with mesenchymal stem cell differently support bone repair in rats. J Mater Sci Mater Med. 2017; 28:35.

9. Hayashi K, Ochiai-Shino H, Shiga T, Onodera S, Saito A, Shibahara T, Azuma T. Transplantation of human-induced pluripotent stem cells carried by self-assembling peptide nanofiber hydrogel improves bone regeneration in rat calvarial bone defects. BDJ Open. 2016; 2.

10. Hsiao H, Yang S, Brey EM, Chu I, Cheng M. Hydrogel Delivery of Mesenchymal Stem Cell–Expressing Bone Morphogenetic Protein-2 Enhances Bone Defect Repair. Plastic and Reconstructive Surgery Global Open. 2016; 4.

11. Wang L, Zhang C, Li C, Weir MD, Wang P, Reynolds MA, Zhao L, Xu HH. Injectable calcium phosphate with hydrogel fibers encapsulating induced pluripotent, dental pulp and bone marrow stem cells for bone repair. Materials Science and Engineering: C. 2016; 69:1125-1136.
